# Evaluating endophenotypes for bipolar disorder

**DOI:** 10.1186/s40345-021-00220-w

**Published:** 2021-05-27

**Authors:** Riccardo Guglielmo, Kamilla Woznica Miskowiak, Gregor Hasler

**Affiliations:** 1 Psychiatry Research Unit, Fribourg Network for Mental Health (RFSM), University of Fribourg, Chemin du Cardinal-Journet 3, 1752 Villars-sur-Glâne, Switzerland; 2 Department of Neuroscience, Institute of Psychiatry, Catholic University Medical School, Largo Francesco Vito 1, 00168 Rome, Italy; 3 Copenhagen Affective Disorder Research Centre (CADIC), Psychiatric Centre Copenhagen, Copenhagen University Hospital, Rigshospitalet, Copenhagen, Denmark

**Keywords:** Bipolar disorder, Endophenotype, Neuroinflammation, Neuroimaging, Cognition

## Abstract

**Background:**

Phenotypic heterogeneity is a major impediment to the elucidation of the neurobiology and genetics of bipolar disorder. Endophenotype could help in reducing heterogeneity by defining biological traits that are more direct expressions of gene effects. The aim of this review is to examine the recent literature on clinical, epidemiological, neurobiological, and genetic findings and to select and evaluate candidate endophenotypes for bipolar disorder. Evaluating putative endophenotype could be helpful in better understanding the neurobiology of bipolar disorder by improving the definition of bipolar-related phenotypes in genetic studies. In this manner, research on endophenotypes could be useful to improve psychopathological diagnostics in the long-run by dissecting psychiatric macro phenotypes into biologically valid components.

**Main body:**

The associations among the psychopathological and biological endophenotypes are discussed with respect to specificity, temporal stability, heritability, familiarity, and clinical and biological plausibility. Numerous findings regarding brain function, brain structure, neuropsychology and altered neurochemical pathways in patients with bipolar disorder and their relatives deserve further investigation. Overall, major findings suggest a developmental origin of this disorder as all the candidate endophenotypes that we have been able to select are present both in the early stages of the disorder as well as in subjects at risk.

**Conclusions:**

Among the stronger candidate endophenotypes, we suggest circadian rhythm instability, dysmodulation of emotion and reward, altered neuroimmune state, attention and executive dysfunctions, anterior cingulate cortex thickness and early white matter abnormalities. In particular, early white matter abnormalities could be the result of a vulnerable brain on which new stressors are added in young adulthood which favours the onset of the disorder. Possible pathways that lead to a vulnerable brain are discussed starting from the data about molecular and imaging endophenotypes of bipolar disorder.

## Background

The diagnosis of Bipolar Disorder (BD) is based on clusters of symptoms and characteristics of clinical course that do not necessarily describe homogenous disorders but that rather reflect final common pathways of different pathophysiological processes involving genetic and environmental contributors. In addition, the boundaries between BD and schizophrenia and between BD and recurrent major depression are not as distinct as assumed in the past. Specifically, heterogeneity implicit in the current classification schema is a reason for the limited success of clinical studies, at the levels of treatment, neurobiology, and genetics. In contrast to other branches of medicine, psychiatry suffers from a diagnostic and classification system that is not based on pathophysiology and etiology, being dependent on nosological tradition, expert consensus, psychometric reliability, and clinical utility. Dissecting psychiatric macro phenotypes into biologically valid components presumes the ability to make diagnosis more certain, more specific, and more amenable to tailored treatment. In this context, research on endophenotypes finds its value as it has the merit to reduce heterogeneity by defining biological traits that are more direct expressions of gene effects. Moreover, the search of endophenotypes has a prevention potential because they can be used to identify individuals who are at risk of developing a mental disorder. Naturally, endophenotypes could contribute to a clinically useful reclassification that will improve the treatment of mental disorders.

For reminding, the term “endophenotype” was described as an internal, intermediate phenotype (i.e., not obvious to the unaided eye) that fills the gap in the causal chain between genes and distal diseases (Gottesman and Gould 2003) and therefore might help to resolve questions about etiology. The endophenotype concept assumes that the number of genes involved in the variations of endophenotypes representing more elementary phenomena (as opposed to the behavioural macros found in the DSM) and is less than the number involved in producing the full disease (Gottesman and Gould 2003). Endophenotypes provide a means for identifying the “upstream” traits underlying clinical phenotypes, as well as the “downstream” biological consequences of genes (Hasler et al. 2006).

The evaluation of a candidate endophenotype is generally based on the endophenotype criteria concept developed by Gottesman and Gould (2003). However, taking into account the increasing recognition of the importance of epigenetic transformations and developmental factors in the expression of psychiatric phenotypes (Gottesman and Hanson 2005; Hasler et al. 2005), the criterion “state independence” might be particularly difficult to achieve for candidate endophenotypes. Therefore, Hasler et al. (2006) proposed to slightly modify this criterion, emphasizing the role of time and age. Below are the modified criteria:

1. An endophenotype is associated with illness, in the general population.
2. An endophenotype is heritable.
3. An endophenotype is state independent (i.e. manifests in an individual whether or not illness is active) but age-normed and might need to be elicited by a challenge.
4. Within families, endophenotype and illness co-segregate (i.e. the endophenotype is more prevalent among the ill relatives of ill probands compared with the healthy relatives of the ill probands).
5. An endophenotype identified in probands is found in their unaffected relatives at a higher rate than in the general population.

Apart from the above criteria, disease specificity and clinical and biological plausibility have also been discussed as evaluation criteria for endophenotypes (Tsuang et al. 1993), to relate phenotypic definitions to clinically relevant outcomes, to enhance the elucidation of clinically relevant pathophysiological mechanisms (Lavori et al. 2002), and to increase prior probability of utility in genetic studies (Freimer and Sabatti 2003, 2004).

It is important to emphasize that endophenotypes are heritable quantitative measurable biological traits that reflect genetically relevant aspects of the heterogeneous pathophysiology of the disease. As such, endophenotypes are clearly different from diagnostic biomarkers that are evaluated by measures of sensitivity and specificity. This is because it cannot be assumed that the current definitions of psychiatric diseases are biologically valid. Further, a biomarker is not necessarily embedded in the genotype and behavior/psychopathology pathway of a disease as it is the case of an endophenotype.

A number of putative endophenotypes have been proposed for BD, including executive dysfunction, learning and memory impairments, cognitive changes after tryptophan depletion, circadian rhythm instability, dysmodulation of reward and motivation, functional and structural brain abnormalities, sensitivity to psychostimulants and cholinergic sensitivity (Hasler et al. 2006). Unfortunately, few studies systematically assess the criteria needed for a measure to be considered an endophenotype. In this context, well-designed twin, family, and prospective studies evaluating candidate endophenotypes for BD, are still scarce. In twin studies, broader and dimensional diagnostic definitions (e.g., schizophrenia spectrum) might provide higher heritability estimates than narrow diagnostic definitions (e.g., pure schizophrenia). Likewise, in longitudinal studies, broader diagnostic categories (mood disorders spectrum) showed greater stability over time than narrow diagnostic definitions (e.g., pure BD) (Hasler et al. 2006). Thus, relatively broad endophenotypes (brain function endophenotypes, e.g., cognitive performance) might be the most heritable and most appropriate for genetic studies (Hasler et al. 2006) and some authors proposed to relax the disorder-specificity requirement of endophenotypes giving more attention to neurocircuitry activity instead of behaviors as starting points in searches for endophenotypes (Beauchaine and Constantino 2017).

Finally, the National Institute of Mental Health has launched the Research Domain Criteria project (RDoC) in 2009 to explore basic dimensions of functioning that span the full range of human behavior from normal to abnormal. The primary goal of this project is to develop a classification system for mental health disorders that is dimensional (rather than categorical) and that links to neurobiological systems. Endophenotypes could be of great importance in this context. In fact, as they are close to the genes, they could help to identify the genetic basis of core symptom domains of mental disorders. Specifically, they could provide more construct-valid indicators of the six major domains including in the RDoC framework. Moreover, since endophenotypes cut across traditional disorder boundaries, they are consistent with the transdiagnostic approach and emphasis in RDoC on identifying etiological processes that underlie multiple conditions (Miller and Rockstroh 2013).

Here, we presented systematic review articles and meta-analyses, or recent research articles when analytical articles were not available. Our priority was given to longitudinal twin, family, and association studies when available. We did not include endophenotypes that were primarily examined in major depression (e.g., return of depressive symptoms after tryptophan and catecholamine depletion, increased stress sensitivity, or dysfunctions of the hypothalamic–pituitary–adrenal axis). In addition to the specificity criterion, we selected putative endophenotypes on the basis of empirical studies in euthymic patients and in unaffected subjects at substantially increased risk for BD.

**Fig. 1:**
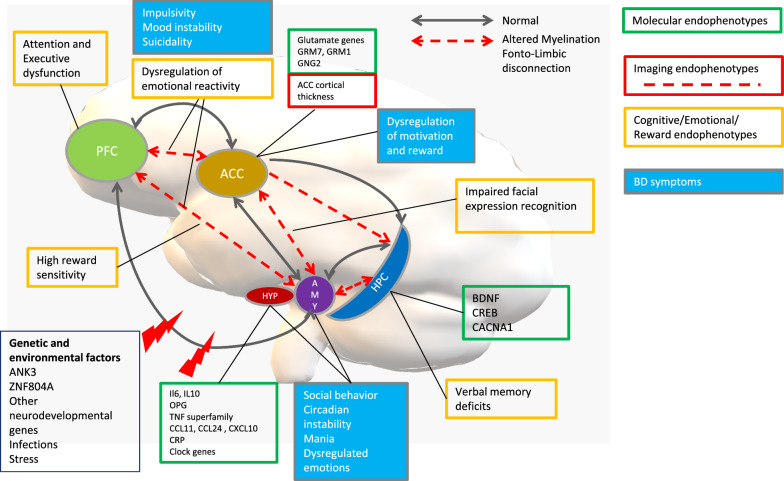
Putative endophenotypes for bipolar disorder. Heuristic model to link the putative endophenotypes (molecular, imaging and cognitive) to distal phenotypes like BD symptoms. Major findings in endophenotype research suggest a developmental origin of BD. This figure shows as the interplay between environmental risk factors like stress and infections, and the genetic susceptibility of different systems like immune, circadian and glutamatergic systems could shape a vulnerable brain on which new stressors are added in young adulthood which favours the onset and the progression of the disorder. *ACC* anterior cingulate cortex, ACNA1 Calcium Voltage-Gated Channel Subunit Alpha1, *ANK3* Ankyrin 3, *AMY* amygdala, *BDNF* Brain Derived Neurotrophic Factor, *CACNA1* Calcium Voltage-Gated Channel Subunit Alpha1, *CCL11* C-C Motif Chemokine Ligand 11, *CCL24* C-C Motif Chemokine Ligand 24, *CXCL10* C-X-C Motif Chemokine Ligand 10, *CRP* C-Reactive Protein, *CREB* CREB Binding Protein, *GRM1* Glutamate Metabotropic Receptor 1, *GRM7* Glutamate Metabotropic Receptor 7, *GNG2* G Protein Subunit Gamma 2, *HPC* hippocampus, *HYP* hypothalamus, *IL6* Interleukin 6, *IL10* Interleukin 10, *OPG* Osteoprotegerin, *PFC* prefrontal cortex, *TNF* Tumor Necrosis Factor, *ZNF804A* Zinc Finger Protein 804A

In this review, we will present majors advances in the field of BD endophenotypes research by proposing the putative BD endophenotypes that best fill the endophenotype criteria. In Fig. 1, we have also enclosed and divided all the main results into molecular, cognitive and imaging endophenotypes. In this manner, we link key neuroanatomical and neurochemical abnormalities to candidate genes and to key behavioural components of BD.

## Molecular endophenotypes

### Altered neuroimmune state

A large body of evidence supports the association between an altered neuroimmune state and BD, although it remains unclear if this reflects a trait or state marker of illness activity or both. The rationale stems from the so-called “sickness behavior,” an interplay between inflammatory states and social and behavioural changes, which includes mood changes, social withdrawal and heightened sensitivity to both positive and negative social stimuli to help an individual to determine which persons might be supportive and which individuals will not and should be avoided, in order to better recruit help and care to facilitate recovery from sickness (Shattuck and Muehlenbein 2016; Dantzer et al. 2008; Eisenberger et al. 2017). Moreover, patients with mood disorders show comorbidities with autoimmune pathologies and subjects suffering of autoimmune diseases present an increased risk of developing BD (Rosenblat and McIntyre 2017; Cremaschi et al. 2017). There is also preliminary pre-clinical evidence suggesting that traditional mood stabilizers modulate neuroinflammation and contrasting clinical results about the action of mood stabilizers on inflammation (Kang et al. 2012; Zhang et al. 2012; Nassar and Azab 2014) as well as some evidence exists about the adjunctive use of drugs with anti-inflammatory properties (i.e. acetylsalicylic, celecoxib and omega-3 fatty acids) for managing BD, in particular bipolar depression (Muneer 2016).

High concentrations of pro-inflammatory cytokines and low concentrations of anti-inflammatory cytokines were reported (Brietzke et al. 2009b) respectively when BD patients in manic and depressive phases are compared to normal controls with a return to normality in the euthymic phase. These illness-related fluctuations suggest that inflammation is a state dependent trait of the acute episodes rather than a trait marker of BD (Cetin et al. 2012).

On the other hand, we have some evidence for an altered neuroimmune state even in euthymic BD patients relative to healthy controls. In particular, euthymic BD patients have been found to display elevated plasma levels of IL-6, osteoprotegerin (OPG) (Hope et al. 2011) and serum levels of IL-10 (Hsu et al. 2014). Euthymic patients have also been found to have elevated levels of tumour necrosis factor [TNF] superfamily molecules, including TNF-like weak inducer of apoptosis [TWEAK] and soluble TNF receptor type 1 [sTNFR1] (Barbosa et al. 2017). Additional evidence indicates increased serum chemokine levels, including CXCL-10, and elevated plasma levels of CCL11, CCL24 and CXCL10 in euthymic patients. In contrast, reduced serum levels of CCL24, and CXCL8 have been found (Brietzke et al. 2009a; Barbosa et al. 2013). Taken together these findings are suggestive of imbalance of cytokines rather than a simple increase. Finally, we have some evidence of abnormally elevated levels of C-reactive protein (CRP) in all phase of BD with a substantial increase during a manic episode and moderate increase in depressive and euthymic phases (Dargel et al. 2015; Fernandes et al. 2016; Evers et al. 2019). Consistent with the research findings on peripheral inflammatory markers, studies using positron emission tomography (PET), revealed increased microglia activation in the brain in euthymic BD patients. Specifically, microglia showed significantly increased activation in the right but not in the left hippocampus of BD patients compared to healthy controls (Haarman et al. 2014). In a subsequent study, an in vivo positive relation was found between microglial activation and neuronal integrity corresponding to a differentiated microglial function where some microglia induce apoptosis while others stimulate neurogenesis (Haarman et al. 2014, 2016). Investigations of the CSF of euthymic BD patients and healthy controls show increased levels of monocyte chemoattractant protein-1 [MCP-1] and chitinase-3-like protein 1 [YKL-40] (Jakobsson et al. 2015), as well as elevated levels of IL-8 and IL-1β (Isgren et al. 2015; Soderlund et al. 2011) in BD. These findings of central inflammatory markers in BD along with the findings about peripheral altered neuroimmune state support the neuroinflammatory hypothesis of BD.

Variance in the inflammatory response seems to be associated with genetic variance in healthy subjects given a strong impact of genetic heritability on inflammatory molecules. A comprehensive study of cytokine production in healthy subjects showed that host genetics plays a significant role in inter-individual variability of cytokine production in response to different types of stimuli with particularly high genetic influence on monocyte derived cytokines, including the IL-1β/IL-6 pathway. In addition, the authors identified 17 new genome-wide significant loci (QTLs) that influence cytokine production, finding biological pathways that contain cytokine QTLs map to pattern recognition receptors (TLR1-6-10 cluster), cytokine and complement inhibitors, and the kallikrein system (Li et al. 2016). Li et al. concluded that monocyte-derived cQTLs are associated to a susceptibility to infections, while Tcell-derived cQTLs overlap with loci associated to autoimmune diseases (Li et al. 2016). The high genetic heritability of inflammatory response is also supported by studies in twins subjects (Wessel et al. 2007; Roederer et al. 2015; Brodin et al. 2015).

There is growing evidence for co-segregation of altered neuroimmune state in families at risk for BD. Significantly increased IL-6 and Brain-derived neurotrophic factor (BDNF) protein levels were found in BD offspring compared to controls (Duffy et al. 2014). The highest levels of both markers were found in those High-Risk (HR) offspring in early clinical stages of the illness compared to later clinical stages. In addition, the genotype of HR individuals influenced the association between risk status and clinical stage of illness development for gene expression. In the early stage of illness development, HR offspring with *BDNF VAL/VAL* genotype had higher mRNA expression levels than HR offspring who were MET carriers (Duffy et al. 2014). These data were not replicated in other studies, possibly due to differences in patients ‘clinical disease stages in sample sizes (Nery et al. 2016; Sanjay et al. 2017). In addition, body mass index (BMI) may have a moderating effect. In fact HR offspring are likely to exhibit elevations in BDNF as a function of increasing weight and moreover a BMI by BDNF interaction in nucleus accumbens (NAc) volume has been found (Mansur et al. 2017).

#### Neuro-immune-developmental hypothesis

The above preliminary evidence for differences in candidate immune and neurotrophic markers over the clinical stages of illness has been confirmed by a temporally dynamic changes of the immune states in offspring of BD patients from adolescence through adulthood in follow-up studies. In comparison to healthy controls, BD offspring showed increased proinflammatory state during adolescence, an anti-inflammatory state during young adulthood and a virtually normalized immune state in adulthood. A background of reduced numbers of circulating CD3+ and CD3+CD4+ T cells is continuously presents, suggesting a partial T cell defect leading to a deregulated immune system (Snijders et al. 2017). In particular, BD offspring showed increased inflammatory gene expression in monocytes, high serum PTX3 levels, but normal CCL2 levels during adolescence. In young adulthood, monocyte activation remained in a lesser degree with serum PTX3 levels remained high, and signs of monocyte migration became apparent through increased CCL2 levels. Finally at adulthood, circulating monocytes lost their activation state, but CCL2 levels remained increased suggesting a stronger migrating activity of these monocytes to the tissues (Mesman et al. 2015). These results were replicated in another study that showed a high inflammatory state in adolescent BD offspring found reduced numbers of Tregs with high expression of pro-inflammatory genes in monocytes. The same studies demonstrated a reduced numbers of T cells capable of producing the proinflammatory cytokines IFN-γ and IL-17 during young adulthood. Finally a stabilisation of these states at adulthood was reported with a reduced expression of pro-inflammatory genes in monocytes (Snijders et al. 2016). Of note, these temporal dynamic changes of the immune state seem to differentiate BD from major depressive disorder (MDD) as some studies suggested that MDD patients share with BD patients an early partial T cell defect but as opposed to BD this immunogical signature becomes more prominent with increasing age, also including an increased immune activation (Grosse et al. 2016).

The finding of a proinflammatory state during adolescence is of particular importance, as the brain is involved in major plastic changes and it is highly vulnerable during this developmental period. Interestingly, an imaging study of youth at risk for BD found altered development of white matter (WM) in the at risk population compared to healthy controls in the corpus callosum and temporal associative tracts, suggesting once again that early altered myelination during development could play a role in the pathophysiology of BD (Versace et al. 2010).

We can therefore conclude that different data support biological and clinical plausibility of an altered neuroimmune state as endophenotype in BD. Cytokines and other inflammatory molecules may pass or act in the CNS at least by 3 different pathways: the “humoral pathway”, which involves cytokine passage through leaky regions in the blood–brain barrier [BBB], “neural pathway”, which involves bind to peripheral afferent nerve fibres such as the vagus nerve, thus stimulating ascending catecholaminergic fibres in the brain, and recently the “cellular pathway”, which involves the trafficking of activated immune cells, typically monocytes, to the brain vasculature and parenchyma has been proposed (Miller and Raison 2016). Inflammatory alterations occur in BD patients as well as in their unaffected relatives (URs) significantly more than in healthy controls either peripherally or centrally in the nervous system and this inflammatory condition can produce negative effects on brain structure and ultimately behavior, especially by early modification of WM structure. In fact, in BD patients it has been shown that WM structure alterations mostly located in the anterior part of the brain, are linked to cytokines levels and T cells percentage (Benedetti et al. 2016; Magioncalda et al. 2018) as well as to the tryptophan degradation cascade, i.e. the increased levels of cytokines reduce the tryptophan-derived serotonin, increasing the production of tryptophan catabolites, which impact on the neurotoxicity (Poletti et al. 2018). Moreover, BD and in general mood disorders have been characterized by accelerated aging. Studies have reported elevated levels of circulating inflammatory cytokines in mood disorders and shorter telomere length, suggestive of accelerating aging (Lindqvist et al. 2015). Consequently, a “telomere-inflammation network” has been proposed to explain a complex interplay between altered inflammatory–immune responses and telomere dynamics in the etiopathogenesis of these disorders. Inflammation and telomere shortening show a bidirectional association: a pro-inflammatory state seems to contribute to aging and telomere dysfunction, and telomere attrition is able to induce low-grade inflammation (Squassina et al. 2019). In line with this, patients with mood disorders show comorbidities with aging-related diseases, such as cardiovascular and respiratory diseases, diabetes, hypertension and cognitive decline which are also characterized by an inflammatory state, that in turn could lead to telomere shortening.

The above findings suggest that an altered neuroimmune state could be a candidate endophenotype for BD and in particular it speaks about a neuro-immune-developmental pathogenesis of BD. In particular, an early proinflammatory state either during pregnancy (Allswede et al. 2020) or adolescence (Mesman et al. 2015; Snijders et al. 2017) may perturb the fetal and young brain development shaping brain structures and in particular WM, followed by a virtually normalized immune state at adulthood with a background of continuously low-grade inflammation responsible of shortening telomere length and accelerated aging, leading to the characteristics symptoms of BD.

### Circadian rhythm instability

Clinical features of BD, such as diurnal variation in mood, early morning awakening, and cyclicity and seasonality of recurrences, have led to speculation that dysregulation of circadian rhythm might be a central mechanism in the pathophysiology of BD. Disturbance of the sleep–wake cycle was found to be the most common prodrome of mania (Jackson et al. 2003; Wehr et al. 1987); experimentally induced sleep deprivation is associated with the onset of hypomania or mania in a considerable portion of patients; and antimanic drugs were shown to stabilize circadian rhythms (Klemfuss 1992; Klemfuss and Kripke 1995).

Twin research in healthy individuals provided evidence for the heritability of circadian clinical characteristics (Dauvilliers et al. 2005) and for a genetic control of the human circadian clock (Linkowski et al. 1993), sleep architecture variables [the amount of slow wave and rapid eye movement (REM) sleep (Linkowski 1999)] and polysomnography profiles during non-REM sleep (De Gennaro et al. 2008). A mutation in a human clock gene, *hPER2*, has been specifically associated with a familial variant of human sleep behavior (Toh et al. 2001), and a polymorphism in the human *CLOCK* gene has been associated with circadian mood fluctuation and illness recurrence in BD (Benedetti et al. 2003).

Findings are conflicting with regard to whereas abnormalities in the circadian rhythm are state independent. Some evidence indicates that the circadian rhythm in euthymic BD patients is persistently unstable (Jones et al. 2005) and can be aggravated with environmental influences, such as weather conditions and seasonal changes (Hakkarainen et al. 2003). Indeed, euthymic BD patients, compared with healthy controls, display trait-like alterations in several circadian features such as sleep time and time in bed, sleep onset latency, melatonin secretion and periods of being awake after sleep onset (Ng et al. 2015; Dallaspezia and Benedetti 2009). In addition, in a systematic analysis of circadian rhythm activity in pedigrees segregating severe BD (BP-I), Pagani et al. (2016) showed that euthymic BD patients slept longer and woke up later compared to their URs. The authors also provided evidence for a genome-wide significant linkage for inter-daily stability, a measure of day-to-day variability of the waveform of activity, near chromosome 12pter. Interestingly, in this genomic region some genes could influence circadian rhythm, including the histone lysine demethylase JARID1a (*KMD5A*) and calcium channel subunit 1C (*CACNA1C*). In contrast, a subsequent study that extended this pedigree and included independent control subjects found no sleep disturbance difference between BD patients, URs and controls, after adjustments for current mood symptoms. This could suggest that state characteristics play a role in circadian abnormalities in BD (Verkooijen et al. 2017).

As for the chronotype, there are some suggestions of a link between BD and evening chronotype, particularly the depressive aspects of BD (Wood et al. 2009). Nevertheless one longitudinal study showed no difference between eveningness and mood state in BD, suggesting that chronotype is independent of mood state and thus a trait marker of the disorder (Seleem et al. 2015).

Lithium, which has been shown to modify the phase and period of circadian rhythms in a variety of species, ranging from unicellular organism and insects to mice and even humans, is a glycogen synthase kinase 3 (*GSK-3*) inhibitor (Gould et al. 2004). Martinek et al. (2001) identified the *Drosophila* orthologue of *GSK-3*, *SHAGGY*, as a component of the circadian cycles. Overexpression of *SHAGGY* lengthened the *Drosophila* free-running circadian cycle. Additionally, a decrease in *SHAGGY* activity resulted in an increase in circadian period length (Martinek et al. 2001), the effect (increase in circadian period) that has been noted in numerous species, including *Drosophila*, after treatment with lithium (Klemfuss 1992; Padiath et al. 2004). Conversely, another study reported that the inhibitors of *GSK-3β* shortened the circadian period of mammalian cells instead of lengthening it (Hirota et al. 2008).

Taken together, these data suggest that the effect of lithium on circadian cycles (Gould and Manji 2002) mediates some of its therapeutic actions in BD, supporting the hypothesis that circadian rhythm instability is etiologically associated with BD and a candidate endophenotype. Finally, preliminary evidence suggests that genetic factors involved in the regulation of the human circadian clock might represent vulnerability factors of BD. Specifically, *GSK3-β* (Benedetti et al. 2004) as well as *CLOCK* (Benedetti et al. 2003; Kripke et al. 2009), *PER3* and *ARNTL* (Nievergelt et al. 2006), *TIMELESS* (Mansour et al. 2005), and *NR1D1 ROR* (Lai et al. 2015) genes all have demonstrated modest associations with BD, supporting a polygenetic heritability.

## Imaging endophenotypes

### Early-onset white matter abnormalities

White matter abnormalities (WMA) are abnormalities in the brain that are seen as bright foci on T2-weighted MRI scans. Most recent techniques include diffusion tensor imaging (DTI) and voxel-based morphometry (VBM). DTI draws maps of the diffusion pattern of water molecules, allowing the detection of microstructural details of normal or altered anatomy of a given region. One of the most used measures of WM DTI studies is fractional anisotropy (FA) which describes the directional selectivity of the random diffusion of water molecules with higher FA values (maximum value is 1.0) are observed along heavily myelinated WM tracts (Kochunov et al. 2012). Voxel-based morphometry (VBM), is an automated MRI technique typically uses T1-weighted volumetric MRI scans and essentially performs statistical tests across all voxels (volumetric picture elements) in the image to identify volume differences between groups (Whitwell 2009). Several studies showed widespread WM disruption in adults BD patients as well as in at-risk subjects.

Evidence of early WMA comes from studies on patients with a first episode BD. A meta-analytic study on this matter found reduced intracranial and WM total volumes in first-episode mania in particular in regions subserving emotional regulation, reward processing and cognitive function (Vita et al. 2009). Several studies have continuously reported decreased fractional anisotropy (FA) in WM tracts connecting circuits implicated in the impaired emotion regulation and reward processing in BD such as prefrontal cortex (PFC) regions with the ventral striatum and amygdala (Schneider et al. 2012). These findings were confirmed by a more recent meta-analysis that found significant total WM volume reduction in first episode BD patients compared to controls. The authors also found some common brain abnormalities present in first-episode schizophrenia or BD with a significant overlap but whole grey matter volume deficits and lateral ventricular enlargement appear to be more prominent in first-episode schizophrenia whereas WM volume reduction seems more prominent in BD (De Peri et al. 2012).

DTI based research has contributed to the notion of structural dysconnectivity between limbic and prefrontal regions in BD. By using DTI techniques, WMA in euthymic BD patients were found in cingulum bundle (Emsell et al. 2013), limbic-striatal, callosal and prefrontal regions (Benedetti et al. 2011b; Emsell et al. 2013; Leow et al. 2013) and in the anterior limb of the internal capsule, anterior thalamic radiation, inferior longitudinal fasciculus, corona radiata, superior longitudinal fasciculus, and uncinate fasciculus (Benedetti et al. 2011a, b; Chaddock et al. 2009). Meta-analyses confirmed the presence of WMA in patients with BD, with the risk of WMA being more than threefold higher in patients with BD than in healthy control populations (Altshuler et al. 1995; Videbech 1997). In particular, DTI meta-analyses studies in BD have shown fractional anisotropy (FA) reduction in clusters located in both anterior and posterior WM areas (Nortje et al. 2013; Vederine et al. 2011), T2 weighted images meta-analytics studies shown increased rates of deep WM hyperintensities (WMH) in BD (Kempton et al. 2008; Beyer et al. 2009), one meta-analysis of voxel based morphometry (VBM) studies shown lower WM concentrations in the left inferior longitudinal fasciculus, left superior corona radiata, and left posterior cingulum (Ganzola and Duchesne 2017). A large and recent meta-analytic study using coordinates, T-maps, and individual MRI data confirmed decreased WM volume in the posterior corpus callosum extending to WM in the posterior cingulate cortex not associated with clinical variables. This suggests that the observed WM decrease is state independent and could be a trait marker of the disease (Pezzoli et al. 2018). Finally, a recent mega-and meta-analysis of the largest DTI dataset of patients with BD, collected via the ENIGMA network, found widespread WMA in BD. Specifically, altered WM connectivity within the corpus callosum and the cingulum were strongly associated with BD diagnosis, suggesting a global profile of illness-related microstructural abnormalities (Favre et al. 2019). However, the findings are not disorder-specific since similar alterations were found in schizophrenia and MDD (Kelly et al. 2018; Wise et al. 2016). Of note, studies suggested that decreased fronto-temporal white matter FA could be applied to differentiate between BD and MDD (Favre et al. 2019; Kelly et al. 2018; Benedetti et al. 2011a; Chen et al. 2016).

WMA as a putative BD endophenotype may have clinical implications. WMA were found to be predictive of lithium and antidepressant response in BD patients (Kato et al. 2000; Bollettini et al. 2015). On the other hand, lithium intake has been associated with neuroprotective effects on WM (Abramovic et al. 2018). In particular, higher FA associated with lithium use could reflect a direct influence of lithium on water diffusion or a beneficial effect on myelination as an increase in axial diffusivity has been linked to the duration of lithium treatment, and influenced by gene variants affecting *GSK-3* via the Wnt/β-catenin and the Akt/CREB pathways (Benedetti et al. 2013; Meffre et al. 2015). Thus, it has been suggested that lithium treatment could counteract the WMA associated with BD (Benedetti et al. 2013).

Findings from twin, family, and association studies have revealed that much of the variability in WM tracts across multiple DTI measures is influenced by genetic factors (Chiang et al. 2011; Jahanshad et al. 2013; Hatton et al. 2018; Lee et al. 2017; Vuoksimaa et al. 2017) and heritability estimates were high in bilateral WM tracts and the corpus callosum (Vuoksimaa et al. 2017). In contrast to genetic influences, shared environmental influences were non-significant for either global or tract-specific influences (Gustavson et al. 2019).

Regarding co-segregation of WMA with the genetic risk of BD, there is compelling evidence that are more frequent in URs compared to controls. Widespread FA reductions and significant lower FA values have been found in BD patients and their URs respect to healthy control subjects particularly in corpus callosum, the dorsal part of the right cingulum bundle, the hippocampal part of the cingulum bundle bilaterally, and the uncinate fasciculus (Sprooten et al. 2013, 2016; Mahapatra et al. 2017; Linke et al. 2020). Of note, a 2-year follow-up study identified similar trajectories of FA reductions for controls and high-risk young adults and failed to find differences in FA among URs of BD patients and healthy controls, suggesting that difference in WM integrity could occur in earlier childhood and be a necessary but not sufficient condition to develop future BD (Ganzola et al. 2017). Another possible explication of the negative results of this follow-up study is that evidence indicates that in individuals at-risk for BD, WMA has been found in specifically brain regions (i.e. superior corona radiata (SCR)/corticospinal tract (CST) and the body of the corpus callosum) whereas changes in other WM tracts seem to be a disease state marker (Linke et al. 2020).

The aetiology of WMA identified in BD is unknown; however, several lines of evidence indicate a potential neurodevelopmental origin, as findings of WMA in individuals at-risk, as well as in early onset BD patients, support the potential involvement of neurodevelopmental mechanisms (i.e. early altered myelination) in the pathophysiology of BD. In support of this hypothesis, genetic studies suggest an involvement of key neurodevelopmental genes as risk factors for WMA in BD (Gurung and Prata 2015). In particular, polymorphisms in *ANK3* that is involved in neurogenesis and in maintaining axonal structure on brain integrity, and in *ZNF804A*, which encodes a zinc finger protein, involved in neurodevelopment and myelin transcription have been associated with BD, and abnormal WM integrity in BD (Squarcina et al. 2017; Gurung and Prata 2015).

Taken together, the data mentioned above suggest WMA and fronto-limbic disconnection as one of the most reliable endophenotype of BD, mediating the relationship between the underlying genetic vulnerability and the clinical expression. WMA in BD may lead to impaired connectivity between cortical and subcortical regions which may have downstream effects leading to core psychopathological features of this disorder, such as impulsivity, processing of emotions and reward, cognitive performance and disrupted night sleep.

### Anterior cingulate cortex and glutamatergic abnormalities

The anterior cingulate cortex (ACC) is the frontal part of the cingulate cortex, with connections to both the limbic system and the prefrontal cortex. The ACC can be divided into two sub regions. The perigenual ACC (pACC) is responsible for processing emotions and regulating the endocrine and autonomic responses to emotions. The dorsal ACC (dACC), also known as the midcingulate cortex, is responsible for cognitive processing, specifically reward-based decision making (Jumah and Dossani 2020).

Lesions of the ventral ACC impair the ability of the autonomic system to respond to emotional stimuli, an inability to experience emotion related to concepts, and inability to use information regarding the probability of aversive social consequences versus reward in guiding social behavior (Damasio et al. 1990). Lesions of the dorsal ACC are associated with attentional deficits and impaired performance on tasks requiring controlled processing (Ochsner et al. 2001).

Volume reductions in the ACC located ventral (subgenual) and anterior (pregenual) to the genu of the corpus callosum have been implicated by numerous studies of mood disorders (Drevets et al. 1997; Harrison et al. 2018; Foland-Ross et al. 2011; Elvsashagen et al. 2013; Lyoo et al. 2006). Specifically, a volume reduction in the left subgenual ACC has been associated with familial MDD and BD by magnetic resonance imaging (MRI) morphometric measures (Drevets et al. 1997) and by post-mortem neuropathological studies, which have shown glial reduction in the corresponding grey matter (Harrison et al. 2018). These findings of volume reduction of ACC are supported by other magnetic resonance imaging studies of the ACC in BD patients which have found reduced cortical thickness in the left anterior cingulate region independent of acute symptoms, suggesting state-independence (Foland-Ross et al. 2011; Elvsashagen et al. 2013; Lyoo et al. 2006).

This reduction in volume exists early in the illness across MDD and BD (Botteron et al. 2002; Hirayasu et al. 1999) but seems to become more pronounced during illness progression, according to preliminary evidence in twins discordant for MDD (Botteron et al. 1999). A volumetric MRI study found reduced subgenual PFC volume in subjects at high familial risk for BD (Drevets 2004). Consistently, another volumetric MRI study comparing URs of patients with BD or schizophrenia showed that anterior ACC abnormalities were specifically associated with genetic risk of BD (McDonald et al. 2004). However, most recent studies on ACC volume and ACC cortical thickness failed to show any significant abnormalities in ACC in URs of BD compared to HC (Hanford et al. 2016; Roberts et al. 2016; Sugranyes et al. 2017; Yalin et al. 2019; Hajek et al. 2008, 2010) with the exception of one study (Sanches et al. 2019). This discrepancy is likely due to small sample size and the presence of lifetime MDD diagnosis in the first-degree relatives of BD of the latter study.

The mechanisms underlying these structural ACC changes remain unclear. However, glutamate may be relevant in BD (Vieta et al. 2018). In particular, the ACC contains abundant concentrations of glucocorticoid receptors that play a major role in attenuating the glucocorticoid response to stress (Diorio et al. 1993). In addition, psychosocial stress enhancement glutamate release and may lead to glutamatergic toxicity (Musazzi et al. 2011).

There is considerable evidence for a high heritability of cortical thickness (Blokland et al. 2012) stress response and cerebral glutamate levels (Legind et al. 2019). Both stress response and glutamate levels have a neurotoxic effect through increases neuronal intracellular calcium levels leading to neuronal cell death or damage (Kritis et al. 2015). Moreover, animal studies found an important role of the Metabotropic glutamate receptor 7 (*GRM7*) in early cortical development. For example, *Grm7* knockdown increases neural progenitor cell (NPC) proliferation, decreases terminal mitosis and neuronal differentiation, leading to abnormal neuronal morphology by interacting with CREB and Yes-associated protein (YAP). Overexpression of *Grm7* along with *Creb* knockdown, or *Yap* knockdown have shown to ameliorate these defects in neurogenesis (Xia et al. 2015). These findings may be relevant for BD since *GRM7* polymorphisms have been associated with the disorder (Noroozi et al. 2019).

Reduced cortical thickness along with glutamatergic abnormalities found in BD and URs bring together the endophenotype criteria of state independent and heritability. This putative endophenotype is not specific for BD; similar abnormalities were also found in MDD and schizophrenia. However, magnetic resonance spectroscopy studies have found disorder-specific changes of the central glutamate system. In BD, Glutamate–Glutamine (Glx) has found to be increased in all mood states (Yuksel and Ongur 2010). In contrast, reduced prefrontal and subcortical GLx has appeared as a consistent finding in MDD. Given the limited reliability and validity of symptom-based diagnostic methods to differentiate between unipolar and bipolar depression, glutamate-related imaging measures have the potential to significantly improve precision and neurobiological validity of mood disorder subtyping.

Taken together, a possible explanation for reduced cortical thickness in BD is a dynamic interplay between the genetic factors influencing brain morphology (i.e. glutamatergic and glucocorticoid system) and environmental factors (i.e. stress) starting from fetal development.

Cortical thickness of Subgenual Anterior Cingulate Cortex (sgACC) is a putative neuroimaging endophenotype for BD because it fills all the endophenotype criteria and it talks about a developmental hypothesis of BD as it could be the result of the underlying susceptibility genes and environmental factors during life acting on the fragility of the cortical structure leading to the clinical expression of BD. Thinning of grey matter of sgACC worth more research as a putative endophenotype of BD as it represents one of the main finding of neuropathological studies with the absence of any contradictory reports up to now (Harrison et al. 2018).

## Cognitive, emotional and reward processing endophenotypes

### Attention and executive functions

Comparative neuropsychological studies in severe psychiatric disorders showed that cognitive impairments in BD patients were similar to those of patients with schizophrenia or MDD with the differences being predominantly quantitative rather than qualitative (Daban et al. 2006; Zaninotto et al. 2015, 2016). In particular executive dysfunction is a key contributor to psychosocial disability, poor occupational functioning and lower quality of life in BD (Goswami et al. 2006; Fountoulakis et al. 2017; Drakopoulos et al. 2020). Executive functions is an umbrella term covering several functions involved in general-purpose control mechanisms, such as planning, inhibition and mental flexibility. Nevertheless, numerous studies and meta-analysis have shown deficits of executive functions across all phase of illness, including euthymia (Kurtz and Gerraty 2009; Bortolato et al. 2015; Bourne et al. 2013; Bora and Pantelis 2015). Specifically, a recent meta-analysis found deficits across set-shifting, inhibition, planning, verbal fluency, working memory, and sustained attention in euthymic BD patients compared to controls (Dickinson et al. 2017).

Individual differences in executive functions are almost entirely genetic in origin as reported by a multivariate twin study of three executive functions (inhibiting dominant responses, updating working memory representations, and shifting attention between task sets) which found that executive functions are influenced by a highly heritable (99%) common factor that goes beyond general intelligence or perceptual speed (Friedman et al. 2008). A highly heritable aspect of executive functions is executive attention, a process that involves dopamine-rich frontal areas including the anterior cingulate (Fan et al. 2001; Swan and Carmelli 2002). In keeping with this, recent results suggest that the dopamine transporter gene (*SLC6A3*) polymorphism influences attentional processes (Kuc et al. 2020). Specifically, individuals with the *9R allele* of the Dopamine transporter gene (*SLC6A3*), when compared to those *10R*, were characterized by less efficient orienting processes and poorer attentional switching (Kuc et al. 2020). Of note, *SLC6A3* polymorphisms have been associated with BD by several studies (Pinsonneault et al. 2011; Greenwood et al. 2006; Douglas et al. 2016). It has been suggested that prefrontal DA and dopaminergic system-related genes play a dominant role in modulating top-down but not bottom-up attention (Schneider et al. 2015). Consistent with this, deficient top-down regulatory mechanisms during anticipation of reward and excessive emotion regulation during anticipation of losses is an important feature of BD patients as shown by fronto-limbic disconnection during reward anticipation in BD (Vai et al. 2019).

Regarding co-segregation of this putative endophenotype, there is compelling evidence that URs of BD patients show reduced executive functions including, executive control (Ferrier et al. 2004; Sepede et al. 2012), sustained attention (Brotman et al. 2009), abstract problem-solving, working memory, interference control (Doyle et al. 2009; Kim et al. 2015), set-shifting (Antila et al. 2007) and response inhibition (Frangou et al. 2005), compared to healthy controls. Consistent with this, meta-analytic evidence indicated worse performance in URs in all cognitive domains studied, including executive functions. Although the effect sizes were small (d < 0.5), they were significantly different from healthy controls for executive function (Arts et al. 2008). This is particularly true for speed dependent measure of executive functions (Bora 2017), a possible marker of abnormality of WM structure as speed of information processing relying on the efficiency of the whole brain network of cortical fibres connections (Wen et al. 2011). Interestingly, some evidence shows that a state of elevated inflammation that in turn led to elevated glutamatergic neurotransmission correlate with poor processing speed (Haroon et al. 2016), implying an interaction between inflammation, increased brain glutamate levels and cognitive impairments in BD subjects.

### Learning and memory

Among cognitive functions, verbal memory deficits were consistently found in BD. Deficits of verbal learning and memory was found in euthymic bipolar patients (Bourne et al. 2013) providing evidence for the state independence of this dysfunction. Verbal learning deficits during the euthymic phase are associated with work disability and poorer clinical and functional outcomes (Burdick et al. 2010; Vieta et al. 2018), representing a major contributor to the overall burden of disability.

Unaffected twins of BD patients also showed reduced short-term and long-term verbal learning and memory (Gourovitch et al. 1999). This corroborates with deficits in verbal long-delay free recall and verbal recognition in URS of BD patients (Sobczak et al. 2002, 2003; Keri et al. 2001). However, evidence from meta-analysis studies in URs is conflicting. Indeed, some studies showed significant deficits in verbal memory (Arts et al. 2008; Bora and Ozerdem 2017; Bora et al. 2009), whereas a recent meta-analysis found no difference in verbal learning and memory between URs of probands with BD and controls (Bora 2017).

In healthy subjects, verbal learning and memory were found to have a particularly high heritability with a genetic component explaining 56% of total variance found in a twin study on memory functions. In particular, this study demonstrated that monozygotic intraclass correlation was significantly larger than the dizygotic correlation for verbal learning and memory but not for response discrimination, learning strategy, and recognition (Swan et al. 1999).

Neurobiological mechanisms that are potentially involved in the synaptic plasticity required for learning and memory include glutamatergic neurotransmission (Bannerman et al. 1995) and changes in gene expression brought about by neurotrophic factors, such as cyclic adenosine monophosphate response element binding protein (CREB) and BDNF (Bourtchuladze et al. 1994; Egan et al. 2003).

The presence of verbal learning and memory deficits in BD patients as well as in first-episode patients and HR subjects, suggest that these deficits are already evident early in BD and that these neurodevelopmental abnormalities have genetic underpinnings.

### Dysregulation of emotion and reward

Individuals with BD are characterized by a dysregulation of the reward system. Heightened incentive motivation and compulsiveness toward reinforced behaviors are characteristic symptoms of the manic phase of BD, whereas loss of interest, lack of reactivity to positive events, and anhedonia are core features of the depressive phase of the disorder. First evidence of a dysregulation of the dopamine reward system came from observations that dopamine agonists induce mania-like behaviour in healthy individuals (Jacobs and Silverstone 1986). Euphoria has been related to amphetamine-induced dopamine release in human ventral striatum (Drevets et al. 2001). Enhanced rewarding effects of psychostimulants in patients with affective illness and induction of mania in individuals with BD has been proposed as a trait-like dysfunction of the dopaminergic system associated with impairment of the brain reward function (Tremblay et al. 2002; Hasler et al. 2006).

Evidence of the heritability of reward abnormalities includes a functional polymorphism of the *COMT* gene that has been associated with the individual variation in the brain response to dopaminergic challenge (Mattay et al. 2003).

Many studies investigating the dysregulation of the behavioural activation system (BAS) and found that individuals along the bipolar spectrum exhibit higher BAS sensitivity than controls even in a euthymic state (Alloy et al. 2008). A prospective study also found that adolescents with no prior history of BD who exhibited an ambitious goal-striving cognitive style at baseline had a greater likelihood and shorter time to first lifetime onset of BD than those without this cognitive style (Alloy et al. 2012). These findings suggest that the high reward sensitivity may be independent of mood state and a potential reward-related endophenotype (Hasler et al. 2006).

Neuroimaging findings point to dysregulation of ventral striatum and mesial prefrontal cortex functions in BD. Increased metabolism in the striatum in both depressed and manic BD patients have been found (Mah et al. 2007; Blumberg et al. 2000), suggesting that elevated metabolic activity in the striatum may be a state-independent illness marker. Interestingly, an fMRI study found a greater ventral striatal and right-sided orbitofrontal (OFC) activity during anticipation, but not outcome, of monetary reward, in euthymic BD patients relative to healthy controls. Interestingly, there was no difference in neural activation between bipolar I and healthy control subjects during anticipation or receipt of monetary loss, suggesting specificity for reward-related fronto-striatal activity as putative trait-like endophenotype in BD (Nusslock et al. 2012; Macoveanu et al. 2020).

Dysmodulation of reward and emotion processing and emotion regulation, including failure to down-regulate emotional reactivity to positive stimuli, may represent a specific endophenotype of BD given evidence of co-segregation of these traits in families at risk for BD. Behavioural studies have found that BD patients and URs compared to healthy controls showed a reduced ability to modulate risk taking in the face of certain types of stressors (Hidiroglu et al. 2013) and increased trait impulsivity and impulsive decision-making (Wessa et al. 2015).

Fronto-limbic disconnection during emotion and reward processing is a putative neuroimaging endophenotype for BD as it could mediate the relationship between the underlying susceptibility genes and the clinical expression of BD (Vai et al. 2014, 2019). Imaging studies have linked the behavioural features to WM integrity in cortico-limbic connectivity with possible deficient top-down regulatory mechanisms during anticipation of reward and deficient emotion regulation during anticipation of losses. In the reward circuitry, youth offspring of parents with BD exhibit altered patterns of frontal activation and ventrolateral prefrontal cortex-striatal functional connectivity than offspring of non-bipolar parents and of healthy parents (Manelis et al. 2016). High-risk offspring had weaker functional connectivity between the pregenual cingulate and the right ventrolateral prefrontal cortex while anticipating rewards than did low-risk offspring, but had a stronger connectivity between these regions while anticipating losses (Singh et al. 2014). These studies joint to aberrant prefrontal activations and connectivity during reward processing in URs.

Compared to health comparison subjects, BD patients and URs showed increased activity of the orbitofrontal cortex and the amygdala, related to heightened sensitivity to reward and deficient prediction error signal (Linke et al. 2012) and in particular WMA coupled with a significant increased number of errors during set shifting and increased risk taking (Linke et al. 2013; Saricicek et al. 2016). This suggests that an altered myelination during development plays a role in the pathophysiology of BD (Caetano et al. 2008).

### Impaired facial expression recognition

Impairments in affective cognition are increasingly recognized as part of the neurocognitive profile and possible treatment targets in BD. Affective cognition may partially reflect neurocognitive functioning in social contexts and moderate the association between neurocognitive impairments and socio-occupational difficulties in BD.

State- and trait-related affective cognitive impairments in BD have been observed across the three phases of the illness and the most consistent findings were trait-related difficulties in facial emotion recognition (Miskowiak et al. 2019). Specifically, a recent systematic review by the International Society of Bipolar Disorder (ISBD) targeting cognition task force, identified global or selective facial emotion recognition deficits in 77% of studies of remitted patients and in 71% of studies of symptomatic BD patients (Miskowiak et al. 2019). Both remitted and symptomatic patients showed difficulties in facial emotion recognition indicating that this may be a trait‐related impairment in BD.

At the same manner, URs exhibit abnormalities at the behavioural and neural levels of emotion processing and regulation, including deficits in the recognition of emotional faces. Several studies showed consistent non-specific deficits in the recognition of facial displays of emotion in URs [for a review see Miskowiak et al. (2017)]. These facial expression recognition problems in URs have been shown to be accompanied by aberrant frontal and/or limbic activation. Unaffected youths have been shown to exhibit decreased amygdala and inferior frontal gyrus response to angry facial expressions (Brotman et al. 2014) and exaggerated amygdala response to fearful (but not happy) faces (Dima et al. 2016; Olsavsky et al. 2012). In contrast, a study of adult URs showed exaggerated amygdala response to happy (but not fearful) faces coupled with increased mPFC reactivity to both happy and fearful faces (Surguladze et al. 2010).

Taken together, there is consistent evidence for aberrant fronto-limbic activity to emotional faces in URs. The discrepancy in the direction the activity changes may be due to different experimental paradigms across studies (i.e., passive viewing vs. task-directed processing of faces), or could indicate age-related differences in individuals at familial risk for BD (Miskowiak et al. 2017).

Notably, a pivotal study including 196 adolescent twins (47 monozygotic and 51 dizygotic pairs) provides the first evidence for heritability of neuroelectric indicators of face emotional processing and suggests that event-related brain potentials (ERPs) components sensitive to emotional expressions can potentially serve as endophenotypes (Anokhin et al. 2010). In particular, the amplitude of the ERP components N240 and P300 showed heritability to emotional response to happy, fearful and neutral faces. This study showed that a substantial proportion of the observed individual variation in these ERP responses can be attributed to genetic factors (36–64% for N240 and 42–62% for P300 components, respectively) (Anokhin et al. 2010).

## Perspectives

Bipolar disorder molecular-related endophenotypes like alteration of the neuroimmune state (Fig. 1, Table 1) showed good evidence regarding endophenotype criteria. Major advances in biological and genetic studies have allowed going beyond the monoamine hypothesis of mood disorders making them more systematic disorders. If on one hand serotonin and dopamine systems retain their importance for symptoms like mood instability, modulation of reward and motivation, cognition, other biological systems have become increasingly important.

**Table 1:** Evaluation of putative endophenotypes for bipolar disorder

Endophenotype	Associated with BD	Heritability	State-independence	Co-segregation	Familial association	Total
Imaging endophenotypes						
White matters abnormalities	+++	+++	+	+	+++	11
Acc cortical thickness	+++	+	+	+	+	7
Cognitive, emotional and reward processing endophenotypes						
Attention and executive dysfunctions	+++	+++	+	++	+	10
Dysregulation of emotion and reward	+++	+	++	+	+	8
Learning and memory	+++	+	+	±	+	6.5
Impaired facial expression recognition	+	+	+	+	+	5
Molecular endophenotypes						
CCL11	+	+	±	+	+	4.5
sTNFR1	+	−	+++	0	0	4
CCL24	++	0	++	−	−	4
CXCL10	++	−	++	0	0	4
BDNF	+	+	±	±	−	3
CRP	+	+	±	±	−	3
TWEAK	++	0	+	0	0	3
IL-10	±	+	±	−	−	2
OPG	+	−	±	0	0	1.5

− one or more studies did not support this finding (with no positive studies), or the majority of studies do not support this finding; ± equal number of studies support this finding and do not support this finding; + at least one study supports this finding and no studies do not support this finding, or the majority of studies support this finding; ++ two or more studies support this finding, and no studies do not support this finding; +++ three or more studies support this finding, and no studies do not support this finding; 0, data not available

Scores: − 0; ± 0.5; + 1

To date, we can assert that we have substantial data about a putative role of an altered neuroimmune state in BD. Research in recent years has elucidated that BD is characterized by an imbalance of inflammatory molecules rather than a simple elevation or reduction of these. Specifically, research on offspring of BD patients suggests that BD could be characterized by a temporal dynamic changes of the immune state: increased proinflammatory state during adolescence, an anti-inflammatory state during young adulthood and a virtually normalized immune state at adulthood suggesting a partial T cell defect i.e. reduced numbers of CD3+ T cells and CD3+CD4+ T helper cells, leading to the deregulated immune system. A neuro-immune-developmental hypothesis of BD could be invoked as an early proinflammatory state either during pregnancy (Allswede et al. 2020) or adolescence (Mesman et al. 2015; Snijders et al. 2017) may perturb the fetal and young brain development shaping brain structures and in particular WM, followed by a virtually normalized immune state at adulthood with a background of continuously low-grade inflammation responsible of shortening telomere length and accelerated aging, leading to the characteristics symptoms of BD.

Because manipulation of the circadian rhythm such as light therapy, phase advance treatment and sleep deprivation can have antidepressant efficacy, circadian abnormalities have been hypothesized to be etiologically associated with affective disorders. Research into circadian rhythm endophenotypes in BD has allowed discovering some genetic factors involved in the regulation of the human circadian clock as vulnerability factors of BD. However, it is not clear if circadian rhythm abnormalities are a trait or state marker of this disorder. In this sense, growing evidence suggests evening chronotype as a putative endophenotype of BD but it warrants further investigation.

Imaging-related endophenotypes (Fig. 1, Table 1) are among those that have received more attention in recent years, thanks to technological advances in the field of neuroimaging. In particular DTI and VBM techniques have allowed confirming early WMA in BD patients and subjects at risk, allowing corroborating the structural dysconnectivity amongst limbic and prefrontal regions as one of the major candidate endophenotypes of BD, mediating the relationship between the underlying genetic vulnerability and the clinical expression of this disorder. The presence of similar pattern of structural dysconnectivity in other major psychiatric disorders like schizophrenia and MDD, confirm that an underlying genetic vulnerability is partially shared among psychotic disorders and mood disorders. However preliminary evidence suggests that WMA and structural dysconnectivity could follow different trajectories in these disorders. In particular studies on untreated first episode BD and schizophrenia suggest that BD patients had decreased FA in cingulum, internal capsule, posterior corpus callosum, tapetum, and occipital WM including posterior thalamic radiation and inferior longitudinal fasciculus/inferior fronto-occipital fasciculus compared to schizophrenic patients. This data suggest that WMA may be intrinsic to BD rather than schizophrenia pathophysiology (Lu et al. 2011). Compared to MDD, studies suggested that decreased fronto-temporal WM FA could be applied to differentiate between BD and MDD (Favre et al. 2019; Kelly et al. 2018; Benedetti et al. 2011a; Chen et al. 2016) and that BD was associated with a greater reduced WM integrity in the left posterior cingulum compared to MDD (Wise et al. 2016).

Targeting the glutamatergic system is one of the last frontier of pharmacological treatment of resistant depression, as esketamine was granted FDA approval in 2019 for MDD and evidence exists for ketamine in bipolar depression (Zarate et al. 2012). Glutamate seems primarily involved in cortical development and it is one of the major players of cortical thickness found in BD patients. The biological role of glutamate in BD could be mediated by interactions between glutamate genetic load (i.e. glutamate genes polymorphisms), hypercortisolemia, stress-induced reduction in neurotrophic factors, and stress-induced reduction in neurogenesis.

BD cognitive-related endophenotypes (Fig. 1, Table 1) have received intensive interest because of their negative impact on quality of life and socio-occupational outcome. In particular, executive dysfunction and verbal memory deficits could be considered as putative endophenotypes of BD. However, evidence suggests that impairments in BD patients were similar to those of schizophrenic or MDD subjects and the differences between these disorders are predominantly quantitative rather than qualitative. It thus seems that BD, MDD and schizophrenia shared a common neurobiological background underlies cognitive deficits making it difficult to research specific genetic mechanisms of BD.

The search for the endophenotypes of BD and more generally of mental disorders remains of considerable importance. In fact, it has implications for the etiology of the disorder, diagnosis and clinical implications. With regards to BD, it is possible to postulate a possible developmental etiology of this disorder. In particular, the latest finding of the research about endophenotypes shows some anomalies already present at the beginning of the disorder and also in young people at risk, confirming this hypothesis. Specifically, early WMA play a central role in the pathophysiology of BD. It is possible to postulate that starting from the fetal period the interplay between environmental risk factors like stress and infections, and the genetic susceptibility of different systems like immune, circadian and glutamatergic systems among others, shape a vulnerable brain. Early WMA could be the result of this vulnerable brain on which new stressors are added in young adulthood which favour the onset of the disorder as the brain does not have the appropriate resilience to resist.

In the long run, the discovery and systematic evaluation of BD endophenotypes, along with identification of specific environmental risk factors, will provide the basis of a new classification system. Such a classification system, based on etiology and pathophysiology, is needed because the improvement of the phenotypic definition of BD will likely facilitate the identification of vulnerability genes and possibly the development of better preventive strategies and treatments.

Research on endophenotypes over the past 30 years has been fruitful, but numerous domains of study deserve further investigation. Animal models and experimental clinical research have to focus on the interplay between genetic and environmental risk factors during development by studying specific brain maturation trajectories. In this sense, a promising field of the research is the relationship between circadian and immune systems to modulate brain maturation in response to external factors such as infection. In fact, several evidences suggest the integration of clock genes, particularly *Bmal1* into the immune control circuitry, allowing organisms to prepare and respond to daily changes in the external environment (Oishi et al. 2017; Nguyen et al. 2013; Early et al. 2018).

*BMAL1* has been linked to BD (Mansour et al. 2006; Nievergelt et al. 2006). It has been shown that *BMAL1* has anti-inflammatory properties in normal conditions, as it rhythmically binds to the promoter regions of *CCL2* and *CCL8*, suppressing their transcription (Nguyen et al. 2013) and his deletion facilitate accumulation of reactive oxygen species and the proinflammatory cytokine, IL-1β (Early et al. 2018). Moreover growing evidence indicates that chemokines and cytokines are key player of normal and pathological brain development (Meyer et al. 2009). The response of the innate immune system to external threats is dependent on time of day. Therefore, a stimulating research objective will be to establish whether the shaping of a brain vulnerable to the development of BD is due to the timing of the interaction between immune and circadian systems genetic load (i.e. polymorphisms) and environmental risk factors.

## Conclusion

The aim of this narrative review was to use a sensitive literature search to provide an integrative synopsis of the topic. Limitations of this strategy include the non-systematic approach of this review selecting a combination of original research, meta-analyses and reviews as sources of information.

Main results of this overview confirm fronto-limbic dissociation linked to WMA as an established imaging endophenotype in BD.

Research about molecular endophenotypes shows a pivotal role of pathways linked to inflammation, circadian, glutamatergic and calcium systems in the pathophysiology and symptoms manifestation of BD. However the lack of studies on co-segregation and particularly familial association in unaffected relatives, do not allow to drawn definitive conclusions.

Findings about cognitive/reward/emotional endophenotypes link BD particularly with attention and executive dysfunction as well as altered reward processing rather than with learning and memory dysfunctions. Impaired facial expression recognition is an interesting and promising field of research, but more investigations are needed.

Even if research about bipolar endophenotypes has been fruitful in the last years, more efforts are needed to perform longitudinal familial studies to better understand possible trajectories of co-segregation and familial associations and to help guide future research. In this context, the definition of endophenotypes in a way that takes developmental and environmental factors into account to detect vulnerability genes is an exciting model for epidemiological research in BD.

## Data Availability

All studies reviewed are published and available.
